# Detailed Distribution of Corneal Epithelial Thickness and Correlated Characteristics Measured with SD-OCT in Myopic Eyes

**DOI:** 10.1155/2017/1018321

**Published:** 2017-05-14

**Authors:** Yanan Wu, Yan Wang

**Affiliations:** Tianjin Eye Hospital and Tianjin Eye Institute, Tianjin Ophthalmology and Visual Science Key Laboratory, Clinical College of Ophthalmology, Tianjin Medical University, No. 4, Gansu Road, Heping District, Tianjin 300020, China

## Abstract

**Purpose:**

To investigate the detailed distribution of corneal epithelial thickness in single sectors and its correlated characteristics in myopic eyes.

**Methods:**

SD-OCT was used to measure the corneal epithelial thickness distribution profile. Differences of corneal epithelial thickness between different parameters and some correlations of characteristics were calculated.

**Results:**

The thickest and thinnest part of epithelium were found at the nasal-inferior sector (*P* < 0.05) and at the superior side (*P* < 0.05). respectively. Subjects in the low and moderate myopia groups have thicker epithelial thickness than those in the high myopia group (*P* < 0.05). Epithelial thickness was 1.39 *μ*m thicker in male subjects than in female subjects (*P* < 0.001). There was a slight negative correlation between corneal epithelial thickness and age (*r* = −0.13, *P* = 0.042). Weak positive correlations were found between corneal epithelial thickness and corneal thickness (*r* = 0.148, *P* = 0.031). No correlations were found between corneal epithelial thickness, astigmatism axis, corneal front curvature, and IOP.

**Conclusions:**

The epithelial thickness is not evenly distributed across the cornea. The thickest location of the corneal epithelium is at the nasal-inferior sector. People with high myopia tend to have thinner corneal epithelium than low–moderate myopic patients. The corneal epithelial thickness is likely to be affected by some parameters, such as age, gender, and corneal thickness.

## 1. Introduction

The corneal epithelium plays a very important role in protecting eyes as it is the outermost layer and in maintaining high optical quality [1, 2] as well. It is found that the epithelium contributed 0.85 D alone in corneal refraction at the 3.6 mm diameter zone [3]. Furthermore, the corneal epithelial thickness is not of homogeneous depth and tends to alter its thickness profile to compensate for irregular corneal stromal surface to get a regular surface [4]. Some corneal surgery and corneal refractive surgery with excimer laser ablation were done directly on corneal epithelium, such as transepithelial photorefractive keratectomy (TransPRK) [5] and phototherapeutic keratectomy (PTK).

Since the corneal epithelium contributes a lot in corneal refraction and it helps in the design of the above surgeries, it is very important to get a better knowledge of the characteristics of corneal epithelial thickness distribution. Previously, a few instruments have been invented and applied to corneal epithelium thickness measurement in vivo, including very high-frequency (VHF) digital ultrasound and confocal microscopy. A few studies on corneal epithelial thickness mapping have been done using very high-frequency (VHF) digital ultrasound and confocal microscopy [6–8]. However, these two techniques have some limitations. They both are invasive devices and need anesthetic. This may increase the risk of corneal infection and decrease the accuracy because of the possible contact-related cornea compression [6, 9, 10]. Since the latest years, SD-OCT has become a promising method to study the corneal epithelial thickness because of its noninvasiveness. It has showed good repeatability and accuracy [11, 12] at the same time. The noncontact, high-speed, and high-resolution characters make SD-OCT a popular device in assessing corneal epithelial thickness. Up to now, only a few research [13–17] could show the corneal epithelium map using noncontact device. This study aimed to figure out the detailed distribution of corneal epithelium.

Furthermore, little knowledge in distinctions of epithelial thickness among different myopia degrees is known. Therefore, with the support of a large sample size, this study aims to investigate the distinction of corneal epithelial thickness in different myopic degrees. The description of corneal epithelial thickness distribution in more detailed parts and correlation between corneal epithelial thickness and various corneal parameters, such as age, corneal thickness, IOP, astigmatism, and corneal front curvature were also analyzed.

## 2. Methods

### 2.1. Subjects

Two hundred and fifteen eyes from 215 healthy subjects (102 women, 113 men) with a mean age of 21.26 ± 4.35 years(18 to 40 years) and mean manifest refraction spherical equivalent (MRSE) of −5.34 ± 2.19 D (ranging from −1.125 D to −12.00 D) participated in this study. Subjects reached a complete ophthalmologic evaluation, including the intraocular pressure (IOP) measurement, best-corrected distance visual acuity (BCVA), slit lamp and ophthalmoscope examination, corneal topography (Pentacam HR, OCULUS GmbH, Wetzlar, Germany), Schirmer I test, and tear break-up time test. Every subject had best-corrected distance visual acuity of 20/25 or better. All measurements were taken without the application of artificial tears or mydriatic eye drops. And the exclusion criteria included suspicious and frank keratoconus, a history of contact lens wear, current or prior ocular pathology, and dry eye disorder. All subjects were informed of the aim of the study, and their consent was obtained at the time of their first clinical visit. This prospective study was performed at the Refractive Surgery Center at the Tianjin Ophthalmology Hospital, Nankai University, and received the approval of the Ethics Committee of our Institution, in accord with the Declaration of Helsinki.

### 2.2. OCT

An ultrahigh resolution SD-OCT (RTVue-100, Optovue Inc., Fremont, CA) was used in this study. The system worked at 830 nm wavelength and had a scan speed of 26,000 axial scans per second. The setting's axial resolution was 5 *μ*m, with an L-Cam lens attached to it, which can take 8 meridional B-scans per acquisition, consisting of 1024 A-scans. A Pachymetry_Cpwr scan pattern centered at the pupil center was used to map the cornea.

The RTVue-100 corneal epithelial thickness mapping and pachymetry software (software version 6.11.0.12) automatically processed the OCT scan to provide the corneal epithelial thickness and pachymetry (corneal thickness) maps, corresponding to a 6 mm diameter area. A well-trained investigator conducted all the measurements, and three repeated measurements were collected and averaged in each case.

### 2.3. Corneal Epithelial Mapping

**Figure 1:**
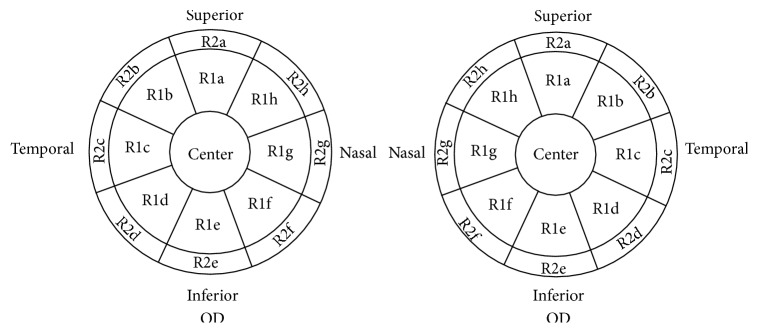
Details of the mapping of corneal thickness and corneal epithelial thickness over the 6 mm diameter cornea from the analyzing report in the set. The analyzing area is divided into three main parts (center, Ring1, and Ring2) and 17 sectors. In Ring1, the sectors were named, respectively, anticlockwise for OD as R1a, R1b, R1c, R1d, R1e, R1f, R1g, and R1h. Similarly, the sectors from Ring2 for OD were named from R2a to R2h. The naming all started from superior to temporal, then inferior to nasal. The left eye map was mirrored.

The analyzing area was two 6 mm diameter disks of corneal thickness and corneal epithelial thickness maps. Each map was divided into 3 zones by diameter: central 2 mm, inner ring from 2 to 5 mm, and outer ring from 5 to 6 mm, according to the set of the analyzing system (Figure 1). The central 2 mm zone was named as center. The 2 to 5 mm zone (named Ring1) and 5 to 6 mm zone (named Ring2) were averagely divided into 8 sectors. The 8 sectors of Ring1 were named anticlockwise for OD as R1a, R1b, R1c, R1d, R1e, R1f, R1g, and R1h. Similarly, the sectors from Ring2 for OD were named from R2a to R2h (Figure 1). The naming all started from superior to temporal, then inferior to nasal. The left eye map was mirrored to the right eye to calculate the difference between the right and left eyes (Figure 1). The average epithelial thickness of each sector was calculated and displayed numerically over the corresponding area. Right eye minus left eye asymmetry (right − left (R-L)) was also calculated (Table 1).

### 2.4. Manifest Refraction Spherical Equivalent (MRSE) Grouping

A set of groups were formed considering the average MRSE of the study population. Group Myopia-L consisted of a low-myopia population, defined as MRSE less than or equal to −3.00 D (*n* = 26), while group Myopia-M was defined as MRSE more than −3.00 D and less than or equal to −6.00 D (*n* = 122), and group Myopia-H consisted of a high-myopia population of MRSE more than −6.00 D (*n* = 67).

### 2.5. Corneal Topography

Anterior segment was imaged with Pentacam (OCULUS GmbH, Wetzlar, Germany). In each acquisition, the rotating Scheimpflug camera captured 50 images automatically and measures 25,000 true elevation points. Due to the good repeatability of this device [18, 19], the acquisition would be applied to the study on condition that the quality specification was “OK.” If not, the acquisition was repeated. The cornea front astigmatism axis (flat) parameter and the mean front corneal surface curvature (Km) were recorded from the Pentacam map.

### 2.6. Statistical Analysis

Statistical Products and Services Solution (SPSS version 20.0, Chicago, Illinois, USA) were used for the statistical analysis. Normal distribution of data was assessed using the Kolmogorov-Smirnov test.

Analysis of variance (ANOVA) was used to compare epithelial thickness in each sector of the 6 mm diameter of cornea and the differences of corneal epithelial thickness in different MRSE groups. The Student's independent-samples *t*-test was used to investigate the difference in epithelial thickness among different parameters, including gender, eye sides, and R-L. Pearson's correlation coefficient was used to compare corneal epithelial thickness to corneal thickness (pachymetry), age, intraocular pressure (IOP), mean front corneal surface curvature (Km), corneal front astigmatism axis (flat), and total eye astigmatism axis. All significant levels were set at *P* < 0.05.

## 3. Results

### 3.1. Corneal Epithelium Distribution

**Figure 2:**
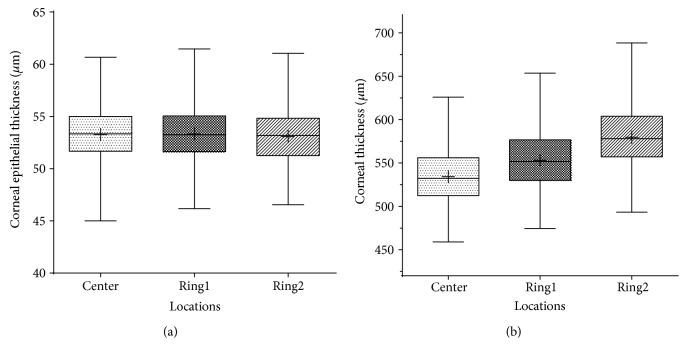
Box plots to show the thickness differences of three locations (center, Ring1, and Ring2) in the corneal epithelial thickness map (a) and corneal thickness map (b). Corneal thickness increased from the center to the periphery (b) while corneal epithelial thickness remained constant (a).

Two hundred and fifteen eyes from 215 subjects were assigned to calculate myopic corneal epithelial thickness and corneal thickness of 17 sectors (Table 2). The central corneal epithelial thickness was 53.26 ± 2.66 *μ*m. The average epithelial thickness of Ring1 and Ring2 were 53.30 ± 2.48 *μ*m and 53.04 ± 2.38 *μ*m, respectively. The central corneal thickness (CCT) was 534.24 ± 29.89 *μ*m. The average of Ring1 and Ring2 were 553.14 ± 30.56 *μ*m and 579.64 ± 31.31 *μ*m, respectively. As Figure 2(a) shows, no statistical difference was found among the center and the two rings of corneal epithelial thickness (*P* = 0.536). The corneal thickness increased gradually from the center to the periphery (*P* < 0.001, Figure 2(b)).

**Figure 3:**
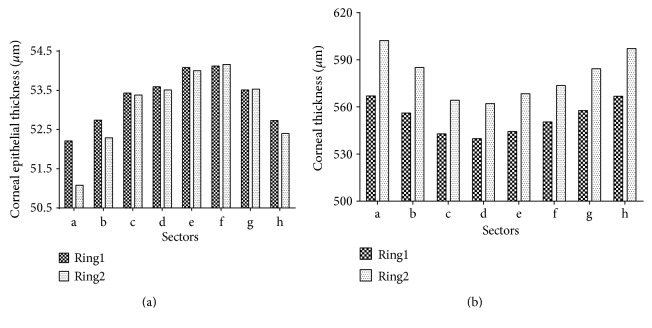
The detailed corneal epithelial thickness (a) and corneal thickness (b) of different sectors in Ring1 and Ring2.

Significant differences in each sector of corneal epithelial thickness value and corneal thickness value were found (Figures 3(a) and 3(b)). As shown clearly in Figure 3(a), R1e and R1f were remarkably larger than other sectors of Ring1 in corneal epithelial thickness value (*P* < 0.05). Similarly, compared to other sectors of Ring2, R2e and R2f also had larger corneal epithelial thickness value numerously (*P* < 0.05). No statistical difference was found between R1e and R1f, the same with R2e and R2f. As shown in Figure 3(b), R1a and R1h were larger numerously than other sectors of Ring1 in corneal thickness (*P* < 0.001). R2a and R2h were also thicker than other sectors of Ring2 (*P* < 0.001). That is to say, the thickest part of full-thickness cornea is the nasal-superior part.

**Figure 4:**
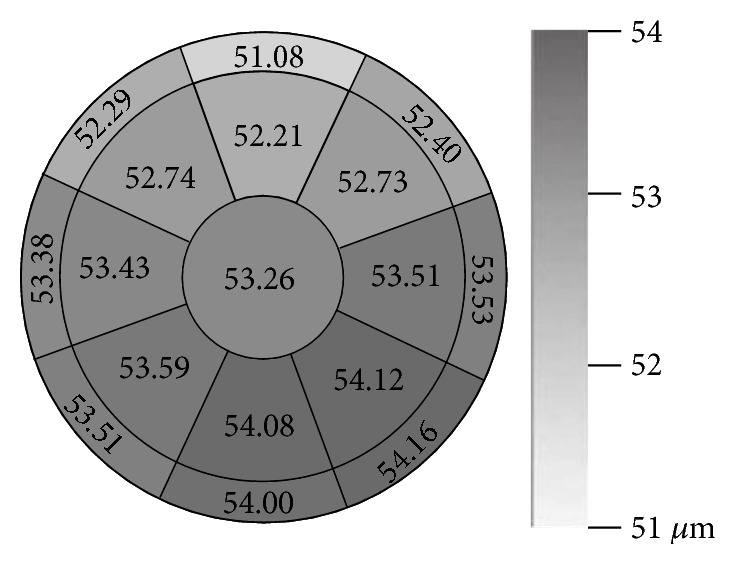
The distribution of corneal epithelial thickness in each sector using color gradations with average thickness on it.

Figure 4 used color gradation to describe the difference in each sector of the corneal epithelial thickness with average thickness on it.

Table 3 showed that there was a weak positive correlation between corneal epithelial thickness and corneal thickness (*r* = 0.148, *P* = 0.031).

### 3.2. Epithelial Thickness Differences in Refraction-Specific Groups

**Figure 5:**
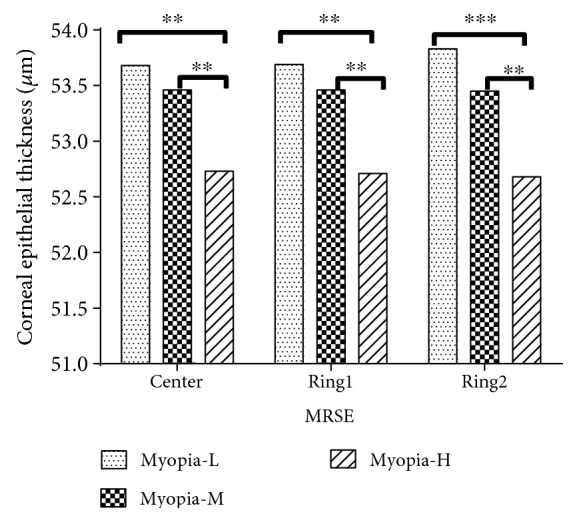
For three locations (center, Ring1, and Ring2), differences of corneal epithelial thickness among different MRSE groups which were divided according to manifest refraction (group myopia-L for less than or equal to 3.00 D, group myopia-M for 3.00 D to 6.00 D, group myopia-H for more than 6.00 D). ^∗∗^ and ^∗∗∗^ indicate *P* < 0.01 and *P* < 0.001, respectively.

As shown in Figure 5, differences of epithelial thickness among different refractions were found. The low and moderate myopia groups (group Myopia-L and Myopia-M) were statistically thicker than high myopia group (group Myopia-H) in the center (0.95 *μ*m, *P* = 0.04; 0.73 *μ*m, *P* = 0.025), Ring1 (0.98 *μ*m, *P* = 0.015; 0.75 *μ*m, *P* = 0.037), and Ring2 (1.15 *μ*m, *P* = 0.002; 0.77 *μ*m, *P* = 0.022). There was no significant difference between Myopia-L and Myopia-M in all locations (*P* > 0.05).

### 3.3. Epithelial Thickness Differences Between the Right And Left Eyes

The differences of corneal epithelial thickness between the right and left eyes were calculated and described in Table 1. The mean difference of R-L in the center, Ring1, and Ring2 were −0.34 *μ*m, −0.35 *μ*m, and −0.34 *μ*m, respectively (*P* > 0.05). Although the average epithelial thickness of the right eye was 0.35 *μ*m thinner than that of the left eye, this difference was not statistically significant (*P* = 0.113).

### 3.4. Epithelial Thickness Differences in Gender-Specific Groups

**Figure 6:**
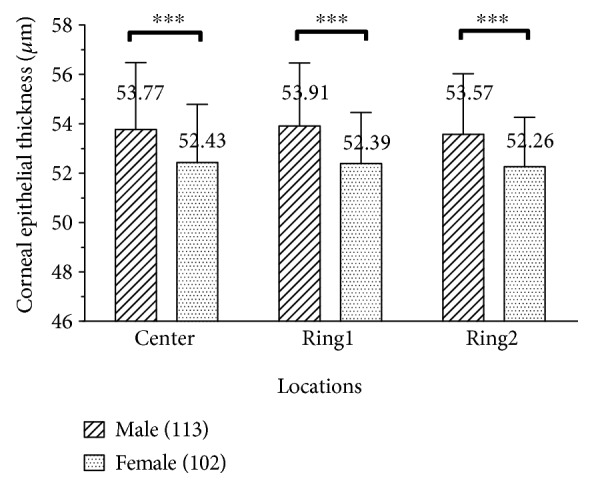
Difference of corneal epithelial thickness between male and female in three locations. ^∗∗∗^ indicates *P* < 0.001.

As shown in Figure 6, the sample was divided into two gender-specific groups: group female (*n* = 102) and group male (*n* = 113). For group female, the average epithelial thickness was 52.43 ± 2.36 *μ*m of the center, 52.39 ± 2.07 *μ*m of Ring1, 52.26 ± 2.00 *μ*m of Ring2, and 52.36 ± 2.05 *μ*m on average. For group male, the average epithelial thickness was 53.77 ± 2.71 *μ*m of the center, 53.91 ± 2.55 *μ*m of Ring1, 53.57 ± 2.46 *μ*m of Ring2, and 53.75 ± 2.48 *μ*m on average. The mean difference between male and female in epithelial thickness value was 1.39 *μ*m (*P* < 0.001).

### 3.5. Correlation with Age, IOP, Corneal Front Curvature, and Astigmatism

In Table 3, there was slight negative correlation between corneal epithelial thickness and age on average (*r* = −0.13, *P* = 0.042). No statistically significant correlation between corneal epithelial thickness and IOP was noted (*r* = −0.006, *P* = 0.934). As for corneal front curvature, there was no statistically significant correlation between corneal epithelial thickness and corneal front curvature (*r* = 0.088, *P* = 0.201). Furthermore, Table 3 shows no significant correlation between corneal epithelial thickness and cornea front astigmatism axis (flat, *r* = −0.051, *P* = 0.456) was noted, nor between corneal epithelial thickness and astigmatism axis in total eye (*r* = 0.043, *P* = 0.527).

## 4. Discussion

A good knowledge of the corneal epithelium distribution may help a lot in many aspects of clinical work, such as screening for keratoconus before corneal refractive surgery [20], fitting contact lens [21, 22], and increasing the accuracy of corneal refractive surgery [23, 24].

The distribution of both corneal thickness and corneal epithelial thickness follow a nonuniform pattern (Table 2 and Figure 3).

The thinnest part of corneal thickness is R1d and R2d, namely, temporal-inferior part. The thickest part is R1a and R1h for Ring1 and R2a and R2h for Ring2, namely, nasal-superior part. The result is in agreement with previously reported values in the use of other evaluation tools [25, 26].

However, the distribution of corneal epithelial thickness is quite different from that of corneal thickness. On the map of corneal epithelial thickness, the thinnest part is R1a for Ring1 and R2a for Ring2. The thickest part is R1e and R1f for Ring1 and R2e and R2f for Ring2. In another word, the thinnest part is the superior and the thickest part is the nasal-inferior. Reinstein et al. [7] reported a similar result in the use of very high-frequency (VHF) digital ultrasound Some previous studies [13, 14, 27] also reported that the inferior side is thicker than the superior, just like this study did.

Concerning the nasal-inferior part to be the thickest part of corneal epithelium over the entire corneal area, one possible explanation of the asymmetry is the eye abrasion caused by the eyelid. Doane [28] reported that the upper eyelid descended fastest at the time it crossed the visual axis. Therefore, the eyelid might be rubbing the corneal epithelium and applied greater forces on the superior cornea than on the inferior part. This might have caused the inferior part of the corneal epithelial thickness to be thicker than the superior part. In this study, weak positive correlation was found between corneal epithelial thickness and corneal thickness (*r* = 0.148, *P* = 0.031). The thickest part of full-thickness cornea is the nasal-superior part. Thus, we postulate that the greater corneal epithelial thickness of the nasal side is related to the corneal thickness. The natural structural difference may be one of the reasons.

It is a limitation here that the tear film was included in the measurement due to the restriction of the machine. Previous study [29] showed that the precorneal tear film was 4.79 ± 0.88 *μ*m on average. This may influence the results of the corneal epithelium distribution, especially the differences between different locations. However, the OCT images were acquired within 5 seconds. We have excluded subjects who had dry eye. We supposed that the tear film was steady during the acquisition process. This would not influence the result too much. Further fundamental research is necessary to search for the reason behind this finding.

The corneal thickness increases gradually from the center to the periphery. However, there is no significant difference among the center, Ring1, and Ring2 in corneal epithelial thickness map in this study. It means that the corneal epithelial thickness remains constant on average from the center to the periphery over the 6 mm diameter area. Tao et al. [30] also reported that the corneal epithelial thickness remained at the same thickness with the use of a different custom-built SD-OCT. In his study, only several points from different locations were acquired.

The low to moderate myopia groups (group Myopia-L and Myopia-M) were statistically thicker than group Myopia-H. According to this, we could deduce that people with high myopia tend to have thinner corneal epithelium than others do. In a clinical study done by Gowrisankaran et al. [31], a correlation between refractive error and blink rate was found. They reported that a refractive error could cause an increasing blink rate (*P* = 0.005). Thus, we deduce that the high myopia patients blink more times than others do. The more frequent eye friction can lead to the thinner epithelial thickness. Furthermore, high myopia is an ocular disease caused by excessive axial elongation. We could also deduce that it may cause thinner corneal epithelial thickness in high myopia eyes. This needs further pathology to confirm. However, some results in previous studies were different. They found that there was no correlation between corneal epithelial thickness and refraction [17, 32]. Further study is needed behind this finding.

Male subjects have thicker corneal epithelial thickness than female subjects do in all three locations (center, Ring1, and Ring2, M-F = 1.39 *μ*m on average, *P* < 0.001). Kanellopoulos and Asimellis [14] also did a similar report of central epithelial thickness. Small differences were noted between male (54.10 ± 3.34 mm) and female (52.58 ± 3.19 mm) subjects. Previous research [33, 34] revealed that gonadal hormones may affect ocular tissue growth. This may cause the difference of corneal epithelial thickness between male and female.

The correlation between corneal epithelial thickness and age is also negative in this study. Kanellopoulos and Asimellis [14] reported that a positive correlation was found between corneal epithelial thickness and age. Reinstein et al. [7] reported that no correlation was found between the two parameters. Different from the other two studies, only young subjects (18–40 years) were recruited in this study. Therefore, the result could be different due to the different age group among different studies.

Since many young patients suffered from myopia, the information provided by this study may to some degree help researchers or others who are interested in corneal epithelial mapping to get more information and develop further research.

Due to the measuring limitation of the SD-OCT, the axial resolution of the system is 5 microns. Because the subjects were healthy except for myopia, their corneal epithelial thicknesses were in the normal range (45–60 microns, 53.26 on average). Therefore, there would not be too much difference numerously among them. Some of the differences observed were lower than 5 microns. Some previous studies [13, 16, 35] also suffered from the same limitation in reporting the results. Maybe the invention of new measuring device with higher resolution will help solve the problem.

To sum up, the profile of the corneal epithelial thickness in myopic eyes was described in this study and confirmed to be nonuniform over the entire cornea. People with high myopia tend to have thinner corneal epithelium than low–moderate myopic patients do. Many factors can be related to the corneal epithelial thickness, such as age, gender, and corneal thickness. Further investigation of the correlation with corneal epithelial thickness might also be needed to expose a specific role for corneal epithelium, such as corneal biomechanics and corneal wounding healing after corneal surgery.

## Figures and Tables

**Table 1:** Distinction of corneal epithelial thickness between the right and left eyes.

		Mean difference (*μ*m)	SEM	Sig.
Right − left (R-L)	Center	−0.34237	0.20142	0.175
	Ring1	−0.35241	0.21179	0.068
	Ring2	−0.34456	0.21583	0.093
	Avg.	−0.35361	0.21945	0.113

**Table 2:** The corneal epithelial thickness and corneal thickness in different locations.

			a	b	c	d	e	f	g	h	Avg.
Epithelial thickness (*μ*m)	Center	Avg.	53.26								
		SD	2.66								
	Ring1	Avg.	52.21	52.74	53.43	53.59	54.08	54.12	53.51	52.73	53.30
		SD	2.62	2.66	2.58	2.56	2.57	2.56	2.60	2.64	2.48
	Ring2	Avg.	51.08	52.29	53.38	53.51	54.00	54.16	53.53	52.40	53.04
		SD	2.68	2.70	2.52	2.54	2.64	2.54	2.53	2.68	2.38
Total thickness (*μ*m)	Center	Avg.	534.24								
		SD	29.89								
	Ring1	Avg.	566.99	556.14	542.89	539.83	544.30	550.44	557.72	566.80	553.14
		SD	31.88	31.55	30.94	30.79	30.39	30.10	30.64	31.33	30.56
	Ring2	Avg.	602.28	585.08	564.26	562.04	568.37	573.62	584.28	597.17	579.64
		SD	33.82	33.06	32.13	31.69	31.39	31.12	31.88	32.34	31.31

**Table 3:** Correlations between corneal epithelial thickness and some parameters.

Location	Age		CT^∗^		Km^∗∗^		Axis-C^∗∗∗^		Axis-T^∗∗∗∗^		IOP	
	*r*	*P*	*r*	*P*	*r*	*P*	*r*	*P*	*r*	*P*	*r*	*P*
Center	−0.11	0.045	0.157	0.021	0.065	0.340	−0.004	0.953	0.033	0.628	0.023	0.741
Ring1	−0.14	0.038	0.148	0.030	0.091	0.185	−0.061	0.373	0.051	0.454	−0.005	0.941
Ring2	−0.11	0.058	0.140	0.040	0.099	0.148	−0.087	0.201	0.041	0.553	−0.037	0.585
Avg.	−0.13	0.042	0.148	0.031	0.088	0.201	−0.051	0.456	0.043	0.527	−0.006	0.934

^∗^CT: corneal thickness; ^∗∗^Km: mean front corneal surface curvature; ^∗∗∗^Axis-C: cornea front astigmatism axis (flat); ^∗∗∗∗^Axis-T: astigmatic axis; IOP: intraocular pressure.
